# Optimizing Vestibular Rehabilitation: From Neuroplastic Mechanisms to Multimodal Therapeutic Strategies

**DOI:** 10.3390/jcm15166359

**Published:** 2026-08-18

**Authors:** Brahim Tighilet, Emna Marouane, Frédéric Xavier, Christian Chabbert

**Affiliations:** 1 Aix Marseille Univ, CNRS, CRPN (Centre de Recherche en Psychologie et Neurosciences-UMR 7077), 13331 Marseille, France; frederic.xavier@etu.univ-amu.fr (F.X.); christian.chabbert@univ-amu.fr (C.C.); 2 Institut de Recherche Équilibre et Vertige (IREV), 30000 Nîmes, France; 3 UNICAEN, INSERM, COMETE, CYCERON, CHU Caen, Normandie Université, 14000 Caen, France

**Keywords:** vestibular compensation, vestibular pharmacology, vestibular rehabilitation, neuroplasticity, gliogenesis, animal model

## Abstract

Peripheral vestibulopathy (PV) is a common disorder that causes dizziness and balance impairment, substantially affecting patients’ quality of life. When symptoms persist, they frequently lead to anxiety, depression, and an increased risk of social isolation. Although central vestibular compensation (CVC) often promotes functional recovery, current pharmacological options remain limited and are primarily aimed at symptom control. Consequently, they should be considered adjuncts rather than alternatives to rehabilitation-based strategies. Vestibular rehabilitation (VR) remains the cornerstone of treatment for promoting functional recovery in patients with PV. Based on the complementary mechanisms of adaptation, substitution, and habituation, VR enhances the central nervous system’s ability to compensate for vestibular deficits by optimizing the integration of visual, proprioceptive, and residual vestibular inputs. Robust evidence from both clinical and preclinical studies has demonstrated its efficacy in improving postural stability, dynamic balance, gaze stabilization, and overall functional performance. Experimental studies using animal models have further highlighted the critical role of active sensorimotor training in ecologically relevant environments for enhancing vestibular compensation. Pharmacological interventions may further facilitate these adaptive processes by modulating the neurobiological mechanisms underlying vestibular compensation, thereby improving responsiveness to rehabilitation. Likewise, emerging neuromodulation approaches, including galvanic vestibular stimulation, have shown promising potential to enhance neural plasticity and augment the effects of rehabilitation. Consequently, combining VR with targeted pharmacological therapies and/or vestibular stimulation techniques may provide synergistic benefits and maximize functional recovery. In conclusion, vestibular rehabilitation should remain the foundation of PV management and be integrated with pharmacological and neuromodulatory approaches within a multidisciplinary therapeutic framework. Further research is needed to optimize rehabilitation protocols, identify the biological determinants of successful vestibular compensation, and develop personalized therapeutic strategies aimed at maximizing functional recovery and improving patients’ quality of life.

## 1. Introduction

Peripheral vestibulopathy (PV) encompasses a group of disorders characterized by impaired peripheral vestibular function, resulting in postural instability, balance impairment, dizziness, and, particularly during the acute phase, episodes of true vertigo. These symptoms markedly interfere with essential daily activities, including standing, walking, head movements, and spatial navigation [1]. In clinical practice, the most prevalent forms of PV include vestibular neuritis, Ménière’s disease, benign paroxysmal positional vertigo, and unilateral or bilateral vestibular hypofunction. When vestibular symptoms become chronic, their impact extends far beyond physical disability. Persistent vestibular dysfunction is frequently associated with anxiety, depressive symptoms, reduced participation in occupational and social activities, and a profound deterioration in health-related quality of life [2,3,4,5]. Owing to its high prevalence in industrialized countries, PV also represents a major public health concern, imposing a considerable socioeconomic burden through healthcare costs, work absenteeism, and loss of productivity [6]. Following peripheral vestibular injury, most patients undergo a spontaneous process of functional recovery known as central vestibular compensation (CVC), which progressively restores postural control and balance over the weeks following symptom onset [7]. However, the extent and rate of compensation vary substantially between individuals, and recovery often remains incomplete, particularly in patients with severe, bilateral, recurrent, or chronic vestibular disorders. For several decades, pharmacological therapies have been used to alleviate vestibular symptoms and, in some cases, to facilitate central vestibular compensation. However, despite their value in symptom management during the acute phase, these treatments have generally failed to provide sustained functional recovery when used as stand-alone interventions. In contrast, vestibular rehabilitation (VR) is now regarded as the cornerstone of treatment for most chronic vestibular disorders. Based on structured, progressive, and task-oriented exercises, VR promotes sensorimotor recalibration by enhancing the integration and reweighting of visual, somatosensory, and residual vestibular inputs, thereby optimizing central compensation and improving postural control, gaze stability, and functional mobility [8,9].

Importantly, vestibular rehabilitation (VR) should not be regarded as a standardized intervention applied uniformly to all patients. Instead, rehabilitation programs should be individualized according to the patient’s underlying vestibular disorder, predominant symptoms, stage of recovery, tolerance to exercise, functional impairments, and rehabilitation goals. Such a personalized approach is particularly important in patients with chronic vestibular disorders and following vestibular surgery, where recovery trajectories are highly heterogeneous and objective clinical or biological biomarkers may help monitor rehabilitation progress, guide therapeutic decision-making, and identify patients at risk of delayed or incomplete compensation [10,11]. Complementary insights into the mechanisms underlying vestibular rehabilitation have been provided by experimental models of peripheral vestibulopathy. These models offer a unique opportunity to evaluate different rehabilitation paradigms under controlled conditions and to investigate their behavioral, anatomical, cellular, and molecular effects. By elucidating the biological substrates of vestibular rehabilitation and central vestibular compensation, preclinical studies may contribute to the development of more effective, mechanism-based rehabilitation strategies while identifying novel pharmacological and neuromodulatory targets capable of enhancing functional recovery.

Accordingly, this review aims to summarize the current understanding of the neuroplastic mechanisms underlying central vestibular compensation and to discuss how these insights may guide the optimization of vestibular rehabilitation. More specifically, we seek to bridge fundamental neurobiological discoveries with clinical practice by highlighting how personalized, multimodal, and progressively adapted rehabilitation strategies can maximize functional recovery and improve the management of patients with peripheral vestibular disorders.

### 1.1. Vestibular Syndrome and Mechanisms of Vestibular Compensation

In most vertebrate species, including humans, vestibular syndrome most commonly results from unilateral damage to the peripheral vestibular apparatus. Bilateral vestibulopathy (BVP), although less frequent, represents a particularly disabling condition characterized by profound postural instability, gait impairment, oscillopsia during head movements, and a markedly increased risk of falls. Following unilateral vestibular injury, vestibular syndrome comprises both static and dynamic deficits. Static deficits, which are present at rest, include postural imbalance resulting from asymmetrical vestibulospinal tone, spontaneous nystagmus, and ipsilesional head tilt. By contrast, dynamic deficits become apparent during movement and are characterized by impaired locomotion, reduced postural stability, and deficient vestibulo-ocular reflex (VOR) function. While static deficits generally resolve within days to weeks through central vestibular compensation, dynamic impairments recover more slowly, often remain incomplete, and may persist for months or even become chronic [12,13]. Beyond these sensorimotor manifestations, vestibular syndrome may also be associated with cognitive, perceptual, and autonomic disturbances, reflecting the widespread central projections and multisystem functions of the vestibular network [14].

Vestibular syndrome follows a characteristic temporal course driven by central vestibular compensation (CVC), a neuroplastic process that underlies the recovery of postural control, balance, and gaze stabilization following vestibular injury. Owing to its well-defined temporal evolution and robust functional outcomes, CVC has become one of the most extensively studied models of injury-induced plasticity in the central nervous system. Comparable compensatory processes are observed in experimental models of peripheral vestibulopathy, which have provided valuable insights into the cellular and molecular mechanisms underlying functional recovery. Traditionally, the restoration of electrophysiological balance between the bilateral vestibular nuclei (VN) has been considered the principal neurophysiological substrate of CVC [15,16,17,18]. This rebalancing is primarily driven by the progressive recovery of spontaneous neuronal activity within the ipsilesional VN, resulting from adaptive changes in the intrinsic membrane properties and excitability of vestibular neurons [19,20,21]. The time course of this electrophysiological recovery varies considerably across species, ranging from approximately one month in cats [22,23,24,25,26] to about one week in guinea pigs [27,28]. Although this vestibulocentric model has long dominated our understanding of central vestibular compensation, the concept that restoration of electrophysiological equilibrium between the ipsilesional and contralesional vestibular nuclei is the sole determinant of functional recovery has progressively been challenged. Increasing experimental evidence indicates that CVC relies on distributed plastic changes involving multiple brain regions and neural networks extending well beyond the vestibular nuclei.

Several experimental observations indicate that the recovery of spontaneous activity within the ipsilesional vestibular nuclei (VN) does not fully account for the time course of functional recovery. Indeed, Ris and colleagues demonstrated a temporal dissociation between the restoration of spontaneous firing in deafferented vestibular neurons and the resolution of several static vestibular deficits [27]. During the acute phase of vestibular syndrome in guinea pigs, namely within the first 90 min following unilateral vestibular injury, animals are already able to partially regain postural stability and maintain a quadrupedal stance despite the complete absence of spontaneous neuronal activity in the ipsilesional VN. A similar temporal mismatch has been described for spontaneous nystagmus. Following unilateral labyrinthectomy in guinea pigs, the intensity of spontaneous nystagmus decreases markedly within the first 10 h after the lesion, whereas spontaneous activity in the ipsilesional VN remains almost completely abolished at the same time point [27]. Comparable observations have been reported in cats, in which postural and locomotor performance returned to baseline approximately one month after unilateral vestibular neurectomy (UVN) [24], despite only partial restoration of spontaneous neuronal activity within the deafferented lateral vestibular nucleus [25,26]. Together, these findings indicate that although the restoration of electrophysiological balance between the ipsilesional and contralesional vestibular nuclei represents a fundamental component of central vestibular compensation, it cannot fully explain the temporal dynamics of functional recovery. Rather, CVC emerges as a distributed process involving multiple complementary mechanisms that operate both within and beyond the vestibular nuclei.

Llinás and colleagues [29] were the first to propose that central vestibular compensation (CVC) should be viewed as a distributed process involving neural networks extending beyond the vestibular system itself. In this framework, the vestibular nuclei (VN) function as central integrative hubs [30], receiving, processing, and redistributing multimodal sensory information from vestibular, visual, proprioceptive, and somatosensory sources. Through this extensive network of connections, the VN play a pivotal role in coordinating vestibulo-ocular, vestibulospinal, and vestibulocollic reflexes that ensure gaze stabilization and postural control during both static and dynamic conditions. Beyond these reflex functions, vestibular information processed within the VN is relayed to widespread spinal, cerebellar, thalamic, cortical, and limbic networks, thereby contributing to higher-order perceptual and cognitive processes, including self-motion perception, body orientation, spatial navigation, and spatial memory. Consequently, functional recovery following vestibular injury is thought to depend not only on the restoration of vestibular neuronal activity but also on sensory substitution mechanisms, whereby intact visual and somatosensory systems progressively compensate for the loss of vestibular input.

Concomitantly, adaptive reorganization occurs throughout the vestibular network. These plastic changes include modifications in the excitability of second-order vestibular neurons, remodelling of synaptic connectivity, and dynamic reweighting of multisensory inputs, collectively contributing to the progressive restoration of vestibular function. Together, these findings support the concept that CVC is a distributed neuroplastic process involving multiple interconnected brain networks. Nevertheless, the vestibular nuclei remain the central substrate of this compensation, as their structural and functional integrity is indispensable for functional recovery to occur [31]. Immediately following peripheral vestibular deafferentation, the primary objective of the ipsilesional vestibular nuclei is to restore a level of neuronal excitability compatible with normal information processing. Achieving this homeostatic state requires a profound reorganization of the deafferented vestibular microenvironment through a coordinated cascade of cellular and molecular events. This adaptive remodelling progressively reshapes the intrinsic properties of vestibular neurons, local synaptic circuits, glial cells, inflammatory responses, and neuromodulatory systems, generating a new functional equilibrium that differs markedly from the pre-lesional state. The mechanisms underlying this extensive neurobiological reorganization are now well characterized and constitute the cellular basis of central vestibular compensation [7,18].

### 1.2. Limitations of Pharmacotherapy for Vertigo

Several randomized clinical trials, systematic reviews, and meta-analyses have questioned the overall efficacy of pharmacological therapies—including betahistine dihydrochloride, acetyl-DL-leucine, and corticosteroids—for the treatment of peripheral vestibular disorders when these conditions are considered as a single, heterogeneous entity. However, the therapeutic benefit of pharmacological interventions is highly dependent on the underlying vestibular pathology, and the efficacy of individual drugs should therefore be evaluated within the context of specific disease entities rather than generalized across all forms of peripheral vestibulopathy.

For example, betahistine remains one of the most widely prescribed treatments for Ménière’s disease and may reduce the frequency and severity of vertigo attacks in selected patients, although the strength of the supporting evidence remains a matter of debate [32]. Similarly, intratympanic gentamicin represents an established therapeutic option for patients with refractory Ménière’s disease, where controlled vestibular deafferentation can effectively suppress recurrent vertigo. In this setting, vestibular rehabilitation plays a key complementary role by facilitating central vestibular compensation and promoting the recovery of postural control, gait stability, and functional mobility following treatment-induced vestibular loss [32].

Overall, pharmacological treatment alone provides only limited functional benefit for most vestibular disorders, and the development of novel therapeutic agents remains relatively modest. Despite their high prevalence, substantial impact on quality of life, and considerable socioeconomic burden, vestibular disorders continue to receive less attention in research funding and public health priorities than many other neurological or sensory disorders. One factor contributing to this situation is the relatively small number of research groups dedicated to the fundamental biology, pathophysiology, and neuropharmacology of the vestibular system. Consequently, vestibular disorders remain underrepresented within the broader scientific and medical communities, limiting awareness of their clinical impact and therapeutic challenges. This lack of recognition may also contribute to delayed diagnosis or misdiagnosis, with many patients undergoing prolonged diagnostic pathways before an accurate vestibular diagnosis is established [33]. Furthermore, the development of new pharmacological therapies is inherently a lengthy process. From target identification and preclinical validation to clinical trials and regulatory approval, the translation of promising experimental compounds into approved therapies generally requires many years before they become available for routine clinical practice.

Beyond their variable therapeutic efficacy, medications commonly prescribed for the symptomatic management of acute vertigo may also influence the effectiveness of vestibular rehabilitation through their adverse-effect profile. Vestibular suppressants, particularly first-generation antihistamines and benzodiazepines, can induce sedation, drowsiness, impaired attention, and reduced physical activity, all of which may limit patients’ active participation in rehabilitation exercises, especially when treatment is prolonged beyond the acute phase. Accordingly, current clinical guidelines recommend restricting their use to short-term symptom control whenever possible, thereby facilitating the early implementation of active vestibular rehabilitation. Importantly, these limitations should not be extrapolated to pharmacological therapies as a whole. Increasing experimental evidence indicates that several pharmacological agents can directly modulate the cellular and molecular mechanisms underlying central vestibular compensation by promoting neuronal plasticity, synaptic remodeling, and adaptive network reorganization. Consequently, rather than representing competing therapeutic approaches, pharmacological treatments and vestibular rehabilitation should be regarded as complementary interventions that converge on shared neuroplastic mechanisms to enhance functional recovery.

Taken together, these observations support the concept that pharmacological therapy alone is unlikely to provide optimal functional recovery for most vestibular disorders. Instead, the greatest therapeutic benefit is likely to arise from multimodal treatment strategies integrating pharmacological therapies with vestibular rehabilitation, each targeting complementary aspects of the compensatory process. While vestibular rehabilitation remains the cornerstone of functional recovery, accumulating preclinical and clinical evidence suggests that appropriately selected pharmacological interventions can facilitate the neuroplastic mechanisms underlying central vestibular compensation and thereby potentiate the effects of rehabilitation within individualized therapeutic programs.

## 2. Vestibular Rehabilitation: An Effective Adjunct and Alternative to Pharmacotherapy

### 2.1. Vestibular Rehabilitation for Patients

#### 2.1.1. General Principles

Vestibular rehabilitation (VR) is a specialized therapeutic intervention designed to improve postural control, gait, gaze stability, and functional mobility in patients with vestibular dysfunction. Following a comprehensive clinical assessment to characterize the vestibular syndrome, establish the underlying diagnosis when appropriate, determine the stage of recovery, and identify the patient’s principal functional impairments, clinicians and physiotherapists with expertise in vestibular rehabilitation develop an individualized rehabilitation program tailored to the specific vestibular disorder. Rather than applying a standardized protocol, VR relies on structured, progressive, and patient-centered interventions adapted to symptom predominance, functional deficits, exercise tolerance, and individual rehabilitation goals [10,11]. Rehabilitation programs typically combine gaze-stabilization, postural control, balance, locomotor, and sensorimotor exercises to promote adaptive motor strategies and optimize the integration of visual, somatosensory, and residual vestibular inputs despite impaired vestibular function. The ultimate objective is to facilitate and maximize central vestibular compensation, thereby restoring functional independence and improving quality of life [8,9]. Over the past two decades, VR has evolved considerably, supported by an expanding body of experimental and clinical evidence. Its efficacy has now been demonstrated across a broad spectrum of vestibular symptoms, particularly in unilateral peripheral vestibular disorders. A recent meta-analysis including 39 studies and 2441 participants confirmed the effectiveness of VR in improving balance, reducing dizziness-related disability, and enhancing functional outcomes in this patient population [34].

Despite the strong evidence supporting its efficacy, the clinical implementation of vestibular rehabilitation (VR) remains highly heterogeneous. Clinicians frequently encounter difficulties in selecting the most appropriate rehabilitation exercises, determining their optimal progression, and adapting treatment protocols to the individual patient’s clinical profile [35]. These challenges underscore the need for personalized, evidence-based rehabilitation strategies tailored to the underlying vestibular disorder, functional deficits, and stage of recovery [36,37,38]. Vestibular rehabilitation is traditionally founded on three complementary mechanisms of recovery: adaptation, substitution, and habituation [39,40,41]. Adaptation-based exercises are designed to promote recalibration of vestibulo-ocular and vestibulospinal reflexes through repeated head movements combined with visual fixation and progressively more demanding multisensory integration tasks. In clinical practice, these exercises typically begin with gaze stabilization on a stationary visual target while the patient performs horizontal or vertical head movements in a seated position. They are then progressively advanced to standing, walking, and increasingly challenging postural conditions by modifying movement velocity, visual environment, support surface, or by incorporating cognitive dual-task paradigms. Adaptation exercises are particularly beneficial for patients presenting with impaired gaze stability, oscillopsia, or deficits of the vestibulo-ocular reflex [38,42,43].

Substitution-based rehabilitation aims to compensate for impaired vestibular function by enhancing the use of alternative sensory inputs. Patients are trained to optimize the integration and reweighting of visual, somatosensory, proprioceptive, and residual vestibular cues in order to improve postural stability, gait, and functional mobility [38]. Typical exercises include standing and walking tasks performed under progressively challenging sensory conditions, such as on compliant or unstable surfaces, with eyes open or closed, during head movements, or in situations that increase reliance on somatosensory information. These interventions are particularly beneficial for patients with postural instability, bilateral vestibular hypofunction, or impaired multisensory integration and sensory reweighting [37,42]. Habituation-based rehabilitation seeks to reduce dizziness and motion-induced symptoms through repeated, controlled exposure to provocative movements or sensory environments. This approach is particularly relevant for patients with motion sensitivity, visual dependence, or persistent visually induced dizziness, in whom complex visual scenes, optokinetic stimulation, or repeated head and body movements provoke or exacerbate symptoms [9,44]. Rehabilitation therefore consists of gradually exposing patients to symptom-provoking stimuli in a graded and individualized manner, allowing symptom intensity to progressively decline through central adaptive mechanisms. Optokinetic stimulation represents a well-established example of this therapeutic principle. Although habituation constitutes an important component of vestibular rehabilitation, it should not be considered its primary mechanism of action. Current evidence indicates that adaptation-based interventions generally produce greater improvements in gaze stabilization, postural control, and overall functional recovery, whereas habituation is primarily indicated for carefully selected patients presenting with visually induced dizziness or motion sensitivity [34,37].

Importantly, these rehabilitation mechanisms are not mutually exclusive but are typically combined and adapted according to each patient’s clinical presentation and stage of recovery. In routine practice, vestibular rehabilitation is usually delivered through repeated short exercise sessions performed daily at home and complemented by supervised sessions with a trained therapist. Most clinical protocols recommend exercises lasting approximately 10–15 min, performed two to three times daily, with progression individualized according to symptom severity, fatigue, exercise tolerance, psychological status, and functional improvement [35,37,38,45]. Such an individualized approach is particularly important because recovery trajectories vary considerably among patients, especially in chronic vestibular disorders and following vestibular schwannoma surgery, where objective clinical or biological biomarkers may help monitor compensation, identify patients at risk of incomplete recovery, and guide rehabilitation adjustments [10,11].

To optimize rehabilitation outcomes, Lacour and Bernard-Demanze proposed ten practical recommendations for the design of vestibular rehabilitation programs [37]. These recommendations emphasize the importance of considering the temporal evolution of vestibular syndrome, exploiting periods of enhanced neuroplasticity, promoting active patient engagement, and prioritizing dynamic, task-oriented, and ecologically relevant exercises that closely reproduce the demands of everyday life. Emerging rehabilitation strategies—including dual-task training, multisensory enrichment, immersive virtual reality, and motivational interventions—may further improve adherence, facilitate functional transfer, and enhance long-term recovery [8]. The psychological dimension of vestibular disorders should also be considered an integral component of rehabilitation. Anxiety, stress, fear of movement, and maladaptive behavioral responses are common in patients with persistent vestibular symptoms and may adversely affect central vestibular compensation, reduce adherence to rehabilitation, and ultimately compromise functional recovery [39,40,41]. Consequently, rehabilitation programs should be tailored not only to the patient’s vestibular deficits but also to their psychological profile, motivation, and tolerance to exercise, particularly in patients with chronic vestibular disorders, visual dependence, or persistent postural-perceptual dizziness.

Taken together, these principles define not only the practical implementation of vestibular rehabilitation but also the neurophysiological rationale underlying its clinical efficacy. By combining adaptation, substitution, and habituation within individualized, progressively challenging rehabilitation programs, VR promotes the neuroplastic processes that restore postural control, gaze stability, and functional mobility following vestibular injury.

#### 2.1.2. Influence of Vestibular Rehabilitation on Posture and Locomotion

Beyond the practical organization of rehabilitation exercises, vestibular rehabilitation (VR) aims to restore postural control, gait stability, and functional mobility by optimizing the integration of multisensory information required for sensorimotor control and by improving the coordination of motor responses [36,42]. These functional adaptations rely on the dynamic interaction of ascending vestibulo-thalamo-cortical, vestibulo-thalamo-striatal, and vestibulo-cerebellar pathways involved in perception, motor planning, and sensorimotor integration, together with descending vestibulospinal pathways that regulate antigravity muscle activity and postural reflexes. Through repeated, task-oriented practice, VR promotes adaptive sensory reweighting, strengthens the coupling between sensory perception and motor output, and facilitates the neuroplastic processes underlying the recovery of both static and dynamic balance [42].

In clinical practice, these neurophysiological adaptations translate into measurable improvements in postural stability, gait performance, gaze stabilization, head–trunk coordination, and the ability to function in visually complex or sensory-conflicting environments. Such functional gains do not depend on a single rehabilitation technique but emerge from the combined effects of gaze-stabilization exercises, postural training, locomotor tasks, sensory conflict paradigms, and dual-task interventions, selected and progressively adapted according to each patient’s impairments and rehabilitation objectives [37,42,43,46]. Accordingly, postural rehabilitation should not be viewed solely as a strategy of sensory substitution but also as a means of promoting adaptive recalibration through more efficient central integration and reweighting of visual, somatosensory, and residual vestibular inputs [43].

The clinical efficacy of VR is supported by numerous studies demonstrating significant improvements in both static and dynamic balance. These benefits are commonly assessed using objective measures such as computerized posturography, gait and functional mobility tests, and validated patient-reported outcome measures evaluating dizziness-related disability and quality of life [8,39,40]. Importantly, these assessments capture clinically meaningful improvements that extend beyond symptom reduction, including increased balance confidence, greater functional independence, and enhanced participation in daily activities. Such multidimensional evaluation is particularly valuable in patients with chronic vestibular disorders or complex recovery trajectories, in whom longitudinal clinical assessment—and, in the future, objective biological biomarkers—may help characterize residual deficits, monitor central vestibular compensation, and further individualize rehabilitation strategies [10,11].

Regular physical activity should be regarded as an essential complement to structured vestibular rehabilitation. More generally, repeated exposure to diverse sensorimotor environments increases the availability and integration of visual, somatosensory, proprioceptive, and locomotor inputs within the central nervous system, thereby promoting postural regulation, adaptive motor control, and functional mobility [35,38,45].

Evidence from studies conducted outside the field of vestibular disorders further supports this concept, demonstrating that long-term engagement in sensorimotor-enriched environments is associated with superior balance performance, enhanced motor adaptability, and greater capacity for sensorimotor learning [47,48]. Although these findings cannot be directly extrapolated to patients with vestibular dysfunction, they provide additional support for incorporating active, ecologically valid, and functionally relevant tasks into vestibular rehabilitation programs. Such approaches are likely to maximize neuroplasticity, facilitate the transfer of acquired skills to everyday activities, and ultimately improve long-term functional outcomes.

### 2.2. The Contribution of Animal Models to the Development of Vestibular Rehabilitation

#### 2.2.1. State of the Art in Animal Studies of VR

Animal models have played a pivotal role in advancing our understanding of vestibular rehabilitation by providing complementary experimental approaches to investigate both the functional consequences of vestibular injury and the mechanisms underlying recovery. Depending on the species and the specific research objectives, vestibular dysfunction has been induced using a variety of models, including unilateral vestibular neurectomy (UVN), unilateral labyrinthectomy (UL), bilateral vestibular lesions, genetically induced vestibular deficits, and age-related vestibular hypofunction.

Large-animal models, particularly cats and non-human primates, have been instrumental in characterizing postural, locomotor, and oculomotor compensation following unilateral vestibular loss, whereas rodent models have provided unique opportunities to investigate the cellular, molecular, and circuit-level mechanisms underlying rehabilitation-induced neuroplasticity. Collectively, these studies have identified key adaptive processes, including changes in neuronal excitability, synaptic remodelling, multisensory integration, and network reorganization.

To reproduce rehabilitation-like conditions, a wide range of experimental paradigms has been developed, including environmental enrichment, treadmill training, voluntary wheel running, task-specific balance and obstacle-crossing exercises, visual deprivation, and controlled sensorimotor manipulations. Together, these complementary models provide valuable translational insights into how active training, sensory substitution, and environmental experience shape central vestibular compensation and promote functional recovery following vestibular injury [49,50].

Over the past four decades, experimental models of vestibular disorders have considerably advanced our understanding of vestibular pathophysiology and the neurobiological mechanisms underlying central vestibular compensation (CVC). These discoveries have contributed to the progressive refinement of rehabilitation strategies aimed at enhancing functional recovery in patients with vestibular disorders. Despite these advances, the cellular and molecular mechanisms through which vestibular rehabilitation (VR) promotes neuroplasticity remain only partially understood. By comparison, the neurobiological effects of rehabilitation have been investigated far more extensively in other neurological disorders, particularly stroke and spinal cord injury, where rehabilitation constitutes a cornerstone of functional recovery. Stroke remains the leading cause of acquired disability in France, affecting approximately 130,000 individuals annually [51], whereas spinal cord injury affects around 2000 people each year, predominantly following traumatic accidents [52]. Consequently, these conditions have benefited from sustained experimental and clinical research efforts. A large body of preclinical evidence has demonstrated that rehabilitation induces multiple forms of structural and functional plasticity after stroke and spinal cord injury. In experimental models, rehabilitation most commonly relies on treadmill-based training paradigms, which stimulate the expression of neurotrophic factors, including brain-derived neurotrophic factor (BDNF) and nerve growth factor (NGF), thereby promoting neuronal survival, synaptic remodelling, axonal sprouting, and neurogenesis [53,54]. Physical training has also been shown to attenuate post-injury neuroinflammation while enhancing axonal remodelling and functional reorganization within motor-related brain regions, including the basal ganglia and cerebellum [55].

In contrast, the neurobiological effects of the motor training paradigms used in vestibular rehabilitation remain much less well characterized. Although experimental models of vestibulopathy have generated important insights into the mechanisms of vestibular compensation, relatively few studies have specifically investigated the cellular and molecular adaptations induced by rehabilitation itself. As a result, the biological basis through which vestibular rehabilitation enhances central vestibular compensation remains considerably less understood than that of rehabilitation in other neurological disorders.

#### 2.2.2. Sensory Deprivation Studies

Importantly, the behavioral deficits observed following unilateral vestibular neurectomy (UVN) result from the vestibular lesion itself rather than from the subsequent sensory deprivation. In these experimental paradigms, sensory deprivation is deliberately introduced as a secondary intervention to reduce or disrupt the compensatory sensory inputs that normally facilitate central vestibular compensation. Depending on the study design, this manipulation may involve restricting sensorimotor activity, limiting locomotion, or removing visual inputs during the critical period of post-lesion recovery. These approaches provide a valuable experimental framework for assessing the contribution of visual, proprioceptive, and motor information to functional recovery. By selectively reducing the availability of these compensatory sensory cues, they consistently demonstrate delayed or impaired recovery of postural control, locomotion, gaze stabilization, and other vestibular functions following unilateral vestibular loss [49,51,52].

Studies using the unilateral vestibular neurectomy (UVN) cat model have provided direct evidence for the essential role of sensorimotor inputs in central vestibular compensation. In this experimental paradigm, vestibular syndrome is induced by unilateral vestibular deafferentation, whereas subsequent sensory deprivation is used as an experimental manipulation to reduce the compensatory sensory information normally available during recovery. Restricting sensorimotor activity during the early post-lesion period—or at the onset of the compensated phase—produces long-lasting impairments in postural recovery compared with control animals, which progressively regain both static and dynamic balance [48].

During the first three days following UVN, control cats preferentially shifted their body weight toward the contralesional side, reflecting the asymmetrical vestibulospinal drive induced by unilateral vestibular loss, a phenomenon also described in rodent models of UVN [55,56]. In contrast, animals subjected to sensorimotor restriction exhibited a prolonged persistence of this postural asymmetry, which remained evident until approximately 12 days after the lesion. Dynamic balance was similarly affected: performance on the rotating beam test was significantly impaired and these deficits persisted well beyond the acute phase, remaining detectable for more than 60 days after UVN [49].

The importance of sensory inputs is further illustrated by studies examining visual deprivation during the critical period of vestibular compensation [57,58]. Animals deprived of visual information exhibited a marked delay in functional recovery, with slower compensation of postural, locomotor, and oculomotor deficits compared with normally reared animals. Together, these findings demonstrate that the availability of visual and sensorimotor information during the early stages of recovery is a critical determinant of the rate and extent of central vestibular compensation.

#### 2.2.3. Balance Training on Primate and Feline Models

A landmark series of studies conducted by M. Igarashi and colleagues between the late 1960s and the early 1980s established many of the experimental foundations of modern vestibular rehabilitation. Using a primate model (squirrel monkey, *Saimiri sciureus*) subjected to unilateral labyrinthectomy (UL), the authors investigated the effects of structured balance training on functional recovery.

In the initial study, animals underwent locomotor training in a rotating cylindrical apparatus for two hours every other day over a six-month period [59]. Dynamic balance was evaluated using a rotating rail paradigm specifically developed for this primate model [60], in which performance was quantified by the animals’ ability to traverse a rail rotating around its longitudinal axis. Although this training protocol did not significantly improve dynamic balance performance, it accelerated the resolution of spontaneous nystagmus, suggesting a beneficial effect on oculomotor compensation [59]. Subsequent refinements of the rehabilitation protocol—including three weeks of preoperative training, daily postoperative exercise sessions, and the implementation of a more sensitive assessment of dynamic balance—demonstrated a clear beneficial effect of rehabilitation on postural and locomotor recovery [61]. In this study, dynamic balance was quantified using a walking corridor equipped with force sensors capable of measuring gait and weight distribution during locomotion [62].

Collectively, these pioneering studies identified several principles that continue to underpin contemporary vestibular rehabilitation. They emphasized the importance of using behavioural tasks that specifically challenge dynamic vestibular function, employing sensitive and clinically relevant outcome measures, providing rehabilitation with sufficient intensity and frequency, and promoting regular physical activity before vestibular injury or surgery whenever feasible. These observations also provided some of the earliest experimental evidence that active sensorimotor training can accelerate central vestibular compensation and improve functional recovery following unilateral vestibular loss.

In the feline unilateral vestibular neurectomy (UVN) model, repeated dynamic training combined with visual stimulation significantly enhances central vestibular compensation (CVC) [58]. In contrast, visual stimulation alone is insufficient to improve dynamic balance. An earlier study in cats subjected to unilateral labyrinthectomy (UL) likewise demonstrated a greater recovery of vestibulo-ocular reflex function in animals housed in an enriched environment and trained to perform jumping tasks than in those maintained under standard housing conditions [63]. Together, these findings emphasize the importance of active training and ecologically relevant rehabilitation paradigms, consistent with the recommendations proposed by Lacour and Bernard-Demanze [37].

#### 2.2.4. Vestibular Rehabilitation in Rodents

In mouse models, the limited number of available studies has reported increased anxiety following balance training in a genetic model of vestibular dysfunction [64] and improved balance performance in aged mice with vestibular hypofunction following postural training [65]. In the genetic model, the rehabilitation protocol consisted of housing animals in an enriched environment designed to promote climbing, exploration, and voluntary physical activity. Hidden food rewards were used to further encourage interaction with the environment. The beneficial effects of this training paradigm were reflected by improved performance in the tail-lift and rotarod tests [64]. Similarly, long-term treadmill training in aged mice with vestibular hypofunction significantly improved dynamic balance [65]. This study also suggested that these functional improvements were associated with synaptic strengthening of vestibular afferent inputs to spinal motor neurons.

Notably, vestibular rehabilitation (VR) remains largely underexplored in rodent models of vestibulopathy. The cellular and molecular mechanisms within the vestibular nuclei (VN) that mediate the beneficial effects of sensorimotor rehabilitation are only beginning to be investigated. A recent study by Zwergal and colleagues, using a rat model of bilateral vestibular lesions, examined the effects of regular voluntary physical activity performed in an enriched environment on both behavioral recovery and functional reorganization of vestibular brain networks [66]. In this model, running wheels were placed in the animals’ home cages, allowing lesioned rats to engage in spontaneous physical activity throughout the recovery period. More recently, our group investigated the effects of a sensorimotor rehabilitation protocol in a rodent model of unilateral vestibulopathy and, for the first time, demonstrated that such training significantly enhances behavioral vestibular compensation [56]. To this end, we developed a rehabilitation paradigm specifically adapted to the unilateral vestibular neurectomy (UVN) rodent model and consistent with current recommendations for vestibular rehabilitation [37]. The objective was to increase motor and somatosensory (tactile and proprioceptive) inputs to the vestibular nuclei during both the critical and compensated phases of vestibular syndrome through treadmill training combined with an obstacle-crossing task. Successful obstacle negotiation requires the integration of visual, proprioceptive, and vestibular information to accurately control limb placement and adapt locomotion to treadmill speed. Furthermore, the progressive increase in task difficulty was designed to implement the principle of adaptation that constitutes one of the fundamental components of vestibular rehabilitation [9,37,38,42].

This tailored rehabilitation protocol produced meaningful improvements in both static and dynamic vestibular function. Improvements in static parameters were observed almost immediately but gradually diminished over time, whereas gains in dynamic performance emerged later and were maintained over the long term. Notably, behavioral outcomes in experimental models are often relatively subtle, and the effects of rehabilitation may therefore be more apparent at the neurobiological than at the behavioral level.

Consistent with these findings, voluntary wheel running following bilateral vestibular loss produced only modest behavioral benefits, primarily reducing body rotations, whereas other locomotor parameters did not differ significantly between the experimental groups [66]. These findings suggest that stereotyped voluntary physical activity alone is insufficient to induce robust and sustained improvements in balance and locomotor function. The authors therefore highlighted the need to develop more sophisticated, task-specific rehabilitation paradigms. In this context, designing preclinical vestibular rehabilitation protocols that incorporate clinically established principles—such as those proposed by Lacour and Bernard-Demanze—appears particularly relevant for improving the translational value of experimental studies.

Despite these encouraging findings, the translational value of animal models must be interpreted with appropriate caution. Although they cannot fully reproduce the clinical heterogeneity of human vestibular disorders—including the diversity of etiologies, age-related multisensory decline, comorbidities, psychological factors, and individual recovery trajectories—and although most experimental studies are performed in young, otherwise healthy animals, they remain indispensable tools for translational vestibular research. Similarly, no single animal model captures the full spectrum of vestibular disorders encountered in clinical practice. Most experimental paradigms involve abrupt and complete vestibular deafferentation, whereas many human vestibular diseases develop progressively or exhibit fluctuating symptoms, as observed in Ménière’s disease and age-related bilateral vestibular hypofunction. Nevertheless, these models faithfully reproduce the principal functional consequences of peripheral vestibular injury while providing unique access to the biological mechanisms that cannot be directly investigated in patients.

In clinical settings, the efficacy of vestibular rehabilitation is primarily evaluated using functional outcome measures, whereas the cellular and molecular mechanisms underlying recovery remain largely inaccessible. In contrast, animal models enable direct correlations between behavioral recovery and changes in neuronal excitability, synaptic remodeling, neuroimmune responses, gliogenesis, and reactive neurogenesis within the vestibular nuclei. Recent experimental evidence further suggests that sensorimotor rehabilitation, like several antivertigo pharmacological interventions, targets common biological pathways—including adaptive microglial remodeling within the deafferented vestibular nuclei—supporting the concept that vestibular rehabilitation is not merely a behavioral intervention but also a biological therapy capable of shaping the neuroplastic processes underlying central vestibular compensation.

Rather than directly predicting clinical outcomes, animal models provide an essential mechanistic framework for identifying novel therapeutic targets, refining rehabilitation strategies, and developing multimodal therapeutic approaches that combine vestibular rehabilitation with pharmacological or neuromodulatory interventions.

### 2.3. Brain Targets of Sensorimotor Rehabilitation

Beyond behavioral outcomes, animal studies suggest that rehabilitation engages plasticity mechanisms at multiple levels of the neuraxis. The sections below summarize the main candidate targets of sensorimotor rehabilitation, spanning spinal circuits, distributed supraspinal networks, and intrinsic plasticity within the vestibular nuclei (VN). The main levels of plasticity involved in vestibular compensation and their potential relevance for vestibular rehabilitation are summarized in Table 1.

#### 2.3.1. Spinal Level

At the spinal level, a recent study demonstrated that long-term physical training in aged mice promotes axonal sprouting of vestibulospinal projections onto spinal motor neurons within the ventral horn, a structural reorganization that was associated with improved balance performance [65]. These findings are consistent with the well-established effects of physical exercise on trophic signaling pathways, including the upregulation of neurotrophic factors that promote axonal sprouting, synaptic remodeling, and reinforcement of neuronal circuits throughout the central nervous system [67,68,69]. By analogy, it is plausible that vestibular rehabilitation protocols involving repeated activation of antigravity muscles and posturo-locomotor synergies similarly strengthen vestibulospinal connectivity after vestibular injury, thereby contributing to more effective postural control and locomotor recovery.

#### 2.3.2. Supraspinal Level

At the supraspinal level, balance training induces widespread structural and functional brain plasticity, even in healthy rodents. Functional MRI studies have demonstrated training-induced changes in both cortical and subcortical regions—including the vestibular nuclei, thalamus, hippocampus, and cerebellum—following only eight days of rotarod training [70]. Similarly, in experimental models of stroke, treadmill training enhances motor recovery through multiple mechanisms, including axonal remodeling within the basal ganglia and motor cortex [53]. Unilateral vestibular deafferentation likewise induces functional reorganization within many of these same brain regions [71], supporting the concept that vestibular injury engages distributed sensorimotor and cognitive networks extending well beyond the vestibular nuclei. In animal models of vestibular loss, longitudinal molecular imaging has further highlighted the contribution of supraspinal plasticity to functional recovery. Using a rat model of bilateral vestibular lesions, Zwergal and colleagues showed by longitudinal PET-CT imaging that regular physical activity was associated with increased synaptic density and enhanced glucose metabolism within the thalamus, a key hub for multisensory integration [65]. Importantly, the extent to which these neuroplastic changes translate into measurable functional improvements is likely to depend on both the sensitivity of behavioral outcome measures and the specificity of the rehabilitation task. Together, these findings support the concept that the nature, complexity, and progressive adaptation of rehabilitation exercises are critical determinants of both the magnitude of neuroplastic remodeling and the transfer of these adaptations to functional recovery.

Finally, in the VN, certain silent neurons become active after deafferentation and are selectively responsive to proprioceptive inputs. These neurons may represent the electrophysiological correlate of sensory substitution mechanisms intrinsic to the VN [72]. However, the underlying plasticity mechanisms, as well as the influence of VR protocols on these mechanisms, remain unknown.

#### 2.3.3. First Clues on the Cellular Correlates of Sensorimotor Rehabilitation in the VN

The vestibular nuclei (VN) constitute the central hub of central vestibular compensation (CVC), and converging evidence indicates that vestibular injury triggers a coordinated cascade of neuroplastic processes within the deafferented VN. These include reactive neurogenesis and gliogenesis, modulation of neuronal excitability, synaptic remodeling, and tightly regulated neuroimmune responses [6,13]. Reactive neurogenesis within the deafferented VN has been demonstrated in the adult cat following unilateral vestibular neurectomy, where newly generated cells survive for at least one month and appear to contribute to functional recovery [73]. More broadly, accumulating evidence supports vestibular reactive neurogenesis as a potential endogenous repair mechanism that promotes central vestibular compensation and the restoration of balance function [6].

Among the molecular pathways implicated in central vestibular compensation, brain-derived neurotrophic factor (BDNF)/tropomyosin receptor kinase B (TrkB) signaling has emerged as a key regulator of neuroplasticity. In the feline unilateral vestibular neurectomy (UVN) model, activation of BDNF/TrkB signaling was shown to facilitate vestibular compensation by promoting reactive neurogenesis and modulating the expression of proteins involved in neuronal excitability, including the potassium-chloride cotransporter KCC2 and GABA receptor subunits [74]. In parallel, neuroimmune responses within the vestibular nuclei are tightly regulated following vestibular deafferentation. Tumor necrosis factor-α (TNF-α) is rapidly upregulated after injury and is followed by transient changes in neuroprotective mediators, including nuclear factor κB (NF-κB) and manganese superoxide dismutase (MnSOD), suggesting that early inflammatory signaling contributes to the establishment of a permissive environment for neural repair and functional recovery [75]. Recent studies have further refined our understanding of the temporal dynamics and cellular origins of glial responses within the deafferented vestibular nuclei, highlighting their potential contribution to the neuroplastic mechanisms underlying central vestibular compensation [76].

Within this framework, a limited but growing number of studies have begun to investigate whether vestibular rehabilitation can modulate these endogenous plasticity programs within the vestibular nuclei (VN). In a rodent model of unilateral vestibulopathy, a sensorimotor rehabilitation paradigm incorporating progressive task difficulty and enhanced somatosensory input was associated with improved recovery of posturo-locomotor function, together with measurable changes in cell survival and glial responses within the deafferented VN [65]. These findings support the hypothesis that rehabilitation promotes functional recovery by (i) facilitating the homeostatic restoration of neuronal excitability within the VN, (ii) strengthening sensory substitution through enhanced integration of non-vestibular inputs, and (iii) modulating neuroimmune and glial responses that contribute to synaptic remodelling [77,78].

Collectively, these observations suggest that vestibular rehabilitation is not merely a behavioral intervention but also a biological therapy capable of influencing coordinated cellular and molecular mechanisms involved in synaptic plasticity, neuronal remodelling, and endogenous repair within the deafferented vestibular nuclei. Nevertheless, only a limited number of studies have specifically examined the cellular and molecular correlates of rehabilitation-induced plasticity in the VN. The first experimental evidence that vestibular rehabilitation can modulate endogenous plasticity in the deafferented vestibular nuclei demonstrated rehabilitation-associated changes in neurogenesis, microgliogenesis, and cell survival [65]. These findings have recently been independently confirmed and extended in a murine model, further supporting the capacity of rehabilitation to influence cell proliferation and glial responses following unilateral vestibular deafferentation [79].

Further elucidating the signalling pathways that couple behavioral training to these endogenous neuroplastic mechanisms represents an important objective for future translational research aimed at optimizing vestibular rehabilitation strategies. However, the precise causal relationships between these cellular adaptations and specific functional improvements remain to be established. Future studies combining standardized, task-specific rehabilitation paradigms with clinically relevant outcome measures will be essential to clarify these mechanisms and to facilitate the translation of experimental findings into evidence-based vestibular rehabilitation protocols [9,37,38,42].

## 3. Pairing Vestibular Rehabilitation with Other Therapies to Optimize Patient Recovery

Taken together, these limitations argue against relying on pharmacotherapy alone and support the rationale for multimodal management strategies. Vestibular rehabilitation (VR) remains the cornerstone intervention to promote sensorimotor recalibration and central vestibular compensation (CVC). However, recovery trajectories depend not only on the content and dose of training, but also on biological factors that may constrain plasticity, including neuroinflammatory responses, stress-related mechanisms, and the availability of neurotrophic support. Accordingly, adjunct interventions may help reduce symptom burden, modulate these constraints, and create more favourable conditions for effective engagement with VR.

Building on the multisite plasticity mechanisms outlined above (Section 2.3 and Table 1), clinical management rarely relies on exercises alone; instead, combined approaches are commonly used to optimize CVC. The following section therefore discusses how pairing VR with complementary interventions may optimize recovery trajectories.

### 3.1. Vestibular Rehabilitation and Pharmacological Treatment

Pharmacological therapy, through the use of antivertigo agents, and vestibular rehabilitation, based on balance training, physical activity, and exercises targeting vestibulo-ocular dysfunction, constitute the two principal therapeutic approaches for the management of vestibular disorders [80]. Although conceptually distinct, both strategies aim to alleviate vestibular symptoms, at least in part by modulating the neuroplastic mechanisms underlying central vestibular compensation (CVC). As a bibliometric indicator, PubMed keyword searches suggest that the scientific literature on vestibular rehabilitation has expanded more rapidly than that on vestibular pharmacology in recent years (Supplementary Figure S1). Vestibular rehabilitation has demonstrated clear clinical efficacy, and research in this field remains highly active, with ongoing efforts to optimize rehabilitation protocols and better understand the neurobiological mechanisms underlying their therapeutic effects [35,42,45,46]. By contrast, no single pharmacological treatment is effective across the full spectrum of vestibular disorders. Most medications appear to provide benefit only in selected vestibular conditions, and for several commonly prescribed antivertigo drugs, both their precise mechanisms of action and their sites of action remain only partially understood [81].

#### 3.1.1. Knowledge Brought from Clinical Practice “…”

In clinical practice, vestibular rehabilitation (VR) and pharmacological therapy are frequently combined, either to reduce symptom burden and facilitate patients’ engagement in rehabilitation or to potentially enhance the neuroplastic mechanisms underlying central vestibular compensation. Betahistine dihydrochloride (BD), a histaminergic agent acting as an H_3_ receptor antagonist and partial H_1_ receptor agonist, remains one of the most widely prescribed antivertigo medications in many countries. Clinical studies evaluating BD, either alone or in combination with VR, have yielded heterogeneous results, largely reflecting differences in patient populations, vestibular disorders, treatment protocols, and outcome measures. For example, Ata et al. compared vestibular rehabilitation with pharmacological treatment in patients with benign paroxysmal positional vertigo (BPPV) and reported greater improvements across several clinical outcomes in the rehabilitation group [82]. In patients with unilateral peripheral vestibular disorders, the addition of high dose betahistine to a vestibular rehabilitation program was associated with greater improvements in disability and postural stability than vestibular rehabilitation alone [83].

Preclinical studies provide a mechanistic rationale for exploring such combined therapeutic approaches. In cats subjected to unilateral vestibular neurectomy, high-dose betahistine—or low-dose betahistine combined with selegiline—accelerated postural recovery and was associated with an increased number of histidine decarboxylase-positive neurons in the tuberomammillary nuclei, consistent with enhanced central histaminergic activity [84]. Because arousal and alertness are thought to play a critical role in functional recovery following vestibular injury [57,66], and because central vestibular compensation involves distributed multisensory and motor networks extending well beyond the brainstem [71], pharmacological modulation may plausibly enhance the neuroplastic processes induced by vestibular rehabilitation at multiple levels of the central nervous system. Nevertheless, well-designed controlled clinical trials are still required to determine whether pharmacological agents exert additive or truly synergistic effects when combined with vestibular rehabilitation.

#### 3.1.2. “…” Should Influence New Approaches in Preclinical Research

Compared with other areas of neuroscience, preclinical vestibular research has only rarely investigated structured rehabilitation-like paradigms in combination with pharmacological interventions. Yet, animal models offer a unique opportunity to disentangle the respective contributions of (i) pharmacological modulation of central vestibular compensation and (ii) rehabilitation-induced sensorimotor recalibration, while simultaneously characterizing the underlying cellular, molecular, and network mechanisms of neuroplasticity.

With regard to pharmacological modulation, the standardized *Ginkgo biloba* extract EGb 761 has been evaluated in a rat model of unilateral labyrinthectomy. Treatment with EGb 761 improved behavioral recovery and was associated with changes in regional cerebral glucose metabolism, as assessed by [^18^F]-FDG micro-PET imaging, particularly within central vestibular and limbic networks [85]. More broadly, longitudinal [^18^F]-FDG micro-PET studies have shown that vestibular compensation is accompanied by dynamic reorganization of whole-brain metabolic networks following unilateral vestibular loss, highlighting the distributed nature of the neuroplastic processes underlying functional recovery [71].

With regard to training-induced effects, locomotor interventions based on voluntary wheel running appear to produce only modest behavioral improvements, even when significant neurobiological changes are observed. In a rat model of bilateral labyrinthectomy, longitudinal dual-tracer PET imaging using [^18^F]-FDG to assess glucose metabolism and [^18^F]UCB-H to quantify synaptic density revealed complex, region-specific patterns of structural and functional brain plasticity. Although wheel-running training resulted in only modest improvements in dynamic balance, it was accompanied by marked neuroplastic changes, particularly within thalamic circuits involved in multisensory integration and vestibular processing [65].

Taken together, these findings highlight two major priorities for translational vestibular research. First, there is a need to develop task-specific, progressively challenging rehabilitation paradigms in animal models that more closely reflect the principles of clinical vestibular rehabilitation. Second, future studies should determine whether pharmacological interventions can enhance rehabilitation-induced neuroplasticity in a phase-dependent manner, particularly during the critical and compensated phases of central vestibular compensation. An important unresolved question is whether drug-induced increases in arousal and locomotor activity can promote greater engagement in rehabilitation, thereby enhancing functional recovery. Likewise, it remains to be established whether combining pharmacological therapy with vestibular rehabilitation produces additive or truly synergistic effects on both behavioral outcomes and imaging-derived biomarkers of neuroplasticity, such as regional brain metabolism and synaptic density, compared with either intervention alone.

### 3.2. Vestibular Rehabilitation and Galvanic Vestibular Stimulation (GVS)

Alongside pharmacological adjuncts, non-invasive neuromodulation has emerged as another candidate strategy to potentiate vestibular rehabilitation (VR). Galvanic vestibular stimulation (GVS) consists of delivering weak electrical currents through surface electrodes—most commonly placed over the mastoid processes—to modulate vestibular afferent firing and downstream vestibular network activity. Initially developed as an experimental probe of vestibular function, GVS is increasingly discussed as a therapeutic tool to reduce balance and gait instability by enhancing the effectiveness of sensorimotor recalibration mechanisms targeted by VR [86].

#### 3.2.1. Stimulation Modalities and Mechanistic Rationale

Classic galvanic vestibular stimulation (GVS) paradigms include (i) direct-current (DC) stimulation, which induces a sustained modulation of vestibular afferent activity; (ii) sinusoidal alternating-current (AC) stimulation, used to elicit frequency-specific vestibular responses; and (iii) noisy or stochastic GVS (nGVS/SVS), in which broadband electrical noise is delivered at subthreshold intensities. The therapeutic rationale for nGVS is largely based on the principle of stochastic resonance, whereby the addition of an optimal level of noise enhances the detection and transmission of weak signals in nonlinear systems. In the vestibular system, this mechanism is thought to amplify residual vestibular inputs and facilitate their central integration during postural and balance tasks. Seminal physiological studies have characterized the effects of GVS across a wide range of stimulation parameters, typically between approximately 0.1 and 4 mA depending on the waveform, electrode montage, and therapeutic objective. These studies demonstrate that GVS primarily modulates otolith-mediated perceptual and postural responses, while additional vestibular effects depend on the specific stimulation paradigm and experimental context [87,88]. Importantly, recent translational reviews have emphasized the considerable heterogeneity of stimulation protocols—including waveform, bandwidth, current intensity relative to perceptual threshold, electrode size, and montage—and have highlighted the need for greater standardization to optimize both the safety and therapeutic efficacy of GVS interventions [89].

Although galvanic vestibular stimulation (GVS) is increasingly being investigated as an adjunct to vestibular rehabilitation, no standardized stimulation protocol has yet been established. Considerable variability persists across published studies with respect to current intensity, waveform characteristics, frequency bandwidth, electrode configuration, stimulation duration, and treatment schedules. This methodological heterogeneity hampers direct comparisons between studies and underscores the need for standardized reporting and harmonized stimulation protocols.

At the same time, the field is increasingly moving toward personalized stimulation strategies, in which GVS parameters are adjusted according to each patient’s perceptual threshold, residual vestibular function, clinical phenotype, and stage of vestibular compensation. This individualized approach is conceptually aligned with the broader evolution of vestibular rehabilitation, where therapeutic interventions are tailored to the patient’s functional impairments, recovery trajectory, and rehabilitation goals. Future research should therefore focus not only on optimizing stimulation parameters but also on establishing patient-specific dosing strategies that maximize therapeutic efficacy while improving the reproducibility, comparability, and clinical translation of GVS-based interventions.

#### 3.2.2. Evidence in Humans: GVS Effects on Balance, Gait, and Vestibular Function

In patients with bilateral vestibulopathy (BVP), several studies have shown that imperceptible noisy galvanic vestibular stimulation (nGVS) can improve postural stability and gait performance, supporting the concept that vestibular noise enhances functional performance when residual vestibular signals are weak. For example, nGVS has been reported to improve dynamic gait stability [90] as well as vestibulospinal function in patients with BVP [91]. Beyond its effects on posturo-locomotor performance, nGVS may also improve perceptual vestibular deficits. Controlled studies have demonstrated enhanced vestibular motion perception during nGVS, particularly in patients with poorer baseline vestibular function [90]. More recently, randomized crossover trials have continued to evaluate the clinical efficacy of nGVS and to identify patient characteristics associated with treatment responsiveness in BVP (e.g., [92]).

Overall, these findings are consistent with the broader concept developed throughout this review: vestibular rehabilitation acts through multisite sensorimotor recalibration and distributed plasticity across vestibular networks, and interventions that enhance the availability or central processing of residual vestibular signals may plausibly augment the efficacy of rehabilitation and improve functional outcomes.

#### 3.2.3. Does GVS Add Value When Paired with VR?

Evidence directly evaluating the combination of vestibular rehabilitation (VR) and galvanic vestibular stimulation (GVS) remains limited but encouraging. Early clinical studies reported that the addition of GVS to a vestibular rehabilitation program enhanced rehabilitation outcomes compared with VR alone [93]. More recently, controlled clinical trials have continued to investigate whether GVS provides incremental benefits when used as an adjunct to conventional vestibular rehabilitation, with some studies reporting additional improvements in postural performance and balance control [94]. However, the available evidence remains heterogeneous with respect to patient populations, outcome measures, stimulation parameters, and the timing of GVS relative to rehabilitation sessions. This methodological variability currently limits the generalizability of the findings and further underscores the need for standardized stimulation protocols and mechanistically informed dosing strategies [89].

#### 3.2.4. Preclinical Support and Broader Neurological Relevance

Animal studies provide mechanistic support for the hypothesis that vestibular electrical stimulation can modulate widespread brain networks involved in gait, arousal, and sensorimotor integration—systems discussed throughout Section 2 and Section 3 of this review. For example, experimental vestibular stimulation has been shown to alter striatal neurochemistry in rats, supporting a functional interaction between vestibular inputs and basal ganglia circuits involved in gait control and Parkinsonian motor deficits [95,96]. In patients with Parkinson’s disease, preliminary studies have also explored the combination of stochastic vestibular stimulation with dopaminergic therapy, demonstrating the feasibility of multimodal therapeutic strategies integrating neuromodulation with conventional pharmacological treatment [97]. Although these studies were not conducted in the context of vestibular disorders, they reinforce the concept that vestibular electrical stimulation can engage distributed multisensory and motor networks—including thalamo-striato-cortical circuits—that are also recruited during vestibular rehabilitation and central vestibular compensation.

Taken together, these findings suggest that GVS, particularly subthreshold noisy GVS (nGVS), represents a promising adjunct to vestibular rehabilitation. By enhancing the functional salience of residual vestibular inputs, reducing symptom burden, and facilitating sensorimotor integration, nGVS may improve patients’ ability to engage in rehabilitation exercises and thereby optimize functional recovery. Nevertheless, its integration into routine clinical practice will require (i) standardized stimulation protocols and reporting guidelines, (ii) identification of responder phenotypes together with the optimal timing of stimulation (e.g., during the critical versus compensated phases of recovery), and (iii) well-designed randomized controlled trials specifically evaluating the incremental benefit of GVS when combined with vestibular rehabilitation compared with rehabilitation alone [86,89].

#### 3.2.5. Emerging Perspectives: Digital, Remote, and Personalized Vestibular Rehabilitation

Beyond pharmacological adjuncts and neuromodulation, the next generation of vestibular rehabilitation is expected to increasingly incorporate digital, immersive, and remotely delivered technologies. However, the clinical value of these innovations will depend less on the technologies themselves than on their ability to deliver rehabilitation strategies grounded in an evolving understanding of the neuroplastic mechanisms underlying central vestibular compensation. Within this framework, virtual reality (VR), augmented reality (AR), telerehabilitation, and wearable sensory-substitution devices should be regarded as complementary tools that can enhance the delivery, intensity, ecological validity, and personalization of vestibular rehabilitation, rather than as substitutes for conventional therapy.

Virtual reality (VR) and augmented reality (AR) are particularly attractive rehabilitation tools because they can recreate visually complex and ecologically relevant environments, provide graded exposure to symptom-provoking stimuli, and potentially enhance patient motivation and adherence. A recent systematic review and meta-analysis suggested that VR/AR-assisted rehabilitation may reduce dizziness-related disability to a greater extent than conventional vestibular rehabilitation alone in patients with peripheral vestibular disorders, although the quality and heterogeneity of the available studies warrant cautious interpretation of these findings [98]. More recently, a randomized controlled trial in patients with acute unilateral vestibulopathy reported that VR-assisted vestibular rehabilitation achieved clinical outcomes comparable to those of conventional therapy, while producing more rapid improvements in the physical component of the Dizziness Handicap Inventory (DHI) and in balance confidence. These findings support the clinical potential of immersive rehabilitation approaches while highlighting the need for greater methodological standardization in future studies [99].

Telerehabilitation also represents a promising extension of vestibular care, particularly for patients with limited access to specialized centers, persistent symptoms, or the need for ongoing monitoring and progression of home-based rehabilitation programs. A 2024 systematic review and meta-analysis provided evidence that telerehabilitation can reduce dizziness severity, disability, and anxiety in patients with vestibular disorders [100]. Similarly, a randomized controlled trial in patients with chronic unilateral vestibular hypofunction demonstrated that an individualized six-week home-based rehabilitation program combined with regular remote supervision improved both objective and patient-reported outcomes, while achieving higher adherence than a customized but unsupervised home exercise program [101]. Collectively, these findings suggest that the effectiveness of telerehabilitation depends less on the digital platform itself than on the delivery of individualized, progressive, and actively supervised rehabilitation rather than generic, unsupervised exercise programs.

Additional emerging technologies include wearable biofeedback and sensory-substitution devices, such as vibrotactile belts and other haptic interfaces that provide real-time directional or postural feedback during standing and walking. These systems are conceptually attractive because they enrich non-vestibular sensory input, facilitate ecological training beyond the clinical setting, and provide task-specific guidance for patients with persistent postural instability or impaired sensory reweighting.

A particularly well-established example is the vibrotactile BalanceBelt, developed for patients with severe bilateral vestibulopathy. This device continuously converts trunk tilt information into vibrotactile signals delivered around the waist, thereby providing an ambulatory sensory-substitution cue during stance and gait. Clinical studies by Kingma and colleagues have shown that the BalanceBelt can improve self-perceived balance and mobility in daily life among a subgroup of patients with severe bilateral vestibular loss who derive insufficient benefit from conventional vestibular rehabilitation, with sustained long-term adherence among responders [102,103].

Nevertheless, the current evidence supporting wearable sensory-substitution systems remains limited and is dominated by feasibility studies, proof-of-concept investigations, small cohorts, and open-label designs. Robust evidence demonstrating durable superiority over well-conducted conventional vestibular rehabilitation is still lacking. At present, these technologies are therefore best regarded as targeted adjuncts or ecological assistive devices, particularly for carefully selected patients with persistent postural instability, impaired spatial orientation, or severe bilateral vestibular loss, rather than as stand-alone rehabilitation strategies.

Future research should determine whether wearable sensory-substitution platforms improve clinically meaningful outcomes—including treatment adherence, fall risk, functional mobility, and participation in daily life—and should identify the patient phenotypes most likely to benefit from haptic or multisensory digital feedback.

Taken together, the available evidence supports immersive, remote, and wearable technologies as promising adjuncts to, rather than replacements for, conventional vestibular rehabilitation. Their principal advantages appear to lie in improving ecological validity, enabling graded exposure to challenging environments, enhancing patient engagement and adherence, and, for selected patients, providing ecological sensory substitution during activities of daily living. However, the current evidence base remains relatively limited and methodologically heterogeneous.

In particular, evidence specifically evaluating augmented reality (AR) remains scarce and is often combined with broader virtual reality (VR) interventions rather than assessed as a distinct therapeutic modality [98,99]. Similarly, telerehabilitation appears to be most effective when it combines individualized home-based exercise programs with structured remote supervision, regular feedback, and progressive adaptation, rather than simply delivering conventional exercises through digital platforms [100,101]. Wearable sensory-substitution devices seem particularly promising for patients with severe bilateral vestibulopathy, in whom functional compensation during everyday activities remains a major unmet clinical need. Nevertheless, their precise role within long-term vestibular rehabilitation strategies and patient management requires further investigation [102,103].

These technological developments should therefore not be viewed as alternatives to the fundamental principles of vestibular rehabilitation, but rather as tools that can enhance their implementation. Their greatest added value is likely to lie in improving ecological validity, increasing opportunities for practice, facilitating symptom monitoring, and enabling a closer alignment between rehabilitation strategies and individual patient characteristics.

From this perspective, future research should aim not only to compare these technologies with conventional rehabilitation, but also to identify the patient phenotypes most likely to benefit from immersive, remotely supervised, wearable, or digitally assisted interventions according to symptom profile, sensory dependence, stage of vestibular compensation, tolerance to cognitive load, and adherence patterns. Ultimately, the future of vestibular rehabilitation will depend less on the emergence of new technologies than on their ability to translate insights gained from experimental models of vestibular disorders into personalized, biologically informed rehabilitation strategies tailored to the needs of individual patients.

## 4. Conclusions

Peripheral vestibular disorders impose a substantial functional burden, and recovery depends critically on the process of central vestibular compensation (CVC). Although pharmacological therapies have been investigated for several decades, currently available agents remain limited in scope and generally fail to provide sustained functional recovery when used alone. In contrast, vestibular rehabilitation (VR) is currently the most effective therapeutic strategy for promoting sensorimotor recalibration, improving postural control, restoring gaze stability, and enhancing functional mobility.

The major challenge is therefore no longer to demonstrate the clinical efficacy of VR, but rather to optimize rehabilitation protocols and elucidate the biological mechanisms that promote or constrain recovery. Experimental studies indicate that rehabilitation engages neuroplastic processes at multiple levels of the central nervous system, including spinal circuits, distributed supraspinal networks involved in multisensory integration, and intrinsic adaptive mechanisms within the vestibular nuclei. Within these structures, changes in neuronal excitability, synaptic remodeling, and glial and neuroimmune responses appear to interact in orchestrating central vestibular compensation. Nevertheless, preclinical research on vestibular rehabilitation remains relatively limited compared with other neurological fields, and the relationships between specific cellular and molecular adaptations and clinically meaningful functional recovery are still far from fully understood.

These considerations support a translational roadmap centered on three major priorities: (i) the development of structured, individualized, task-specific, and progressively challenging rehabilitation paradigms aligned with established clinical principles of vestibular rehabilitation; (ii) the implementation of sensitive and standardized outcome measures integrating behavioral, imaging, and histological endpoints; and (iii) the phase-dependent evaluation of multimodal therapeutic strategies combining vestibular rehabilitation with complementary interventions aimed at reducing symptom burden and creating a more favorable environment for neuroplasticity. Systematic preclinical investigation of these combined approaches will be essential for identifying actionable therapeutic targets, optimizing the timing and dosing of interventions, and ultimately improving functional recovery following vestibular injury.

Another important priority will be the identification of objective biomarkers capable of monitoring both treatment response and the biological processes underlying recovery. Although no validated biomarkers are currently available to assess the efficacy of vestibular rehabilitation, integrating advanced neuroimaging, quantitative functional assessments, wearable technologies, and digital biomarkers with the mechanistic insights provided by experimental models may pave the way for more personalized, biologically informed vestibular rehabilitation strategies.

Future research should also determine how immersive environments, augmented reality (AR)-assisted interventions, and remotely supervised rehabilitation programs can be optimally integrated into phenotype-informed vestibular care while preserving the individualized principles that underpin conventional vestibular rehabilitation. Wearable sensory-substitution devices, such as vibrotactile belts, may also play an important role within this framework, particularly for patients with severe bilateral vestibulopathy, by complementing rehabilitation through continuous ecological sensory support during daily activities rather than acting solely as training-based interventions.

## Figures and Tables

**Table 1:** Main levels of plasticity involved in vestibular compensation and their potential relevance for vestibular rehabilitation.

Level of Plasticity	Main Structures Involved	Main Mechanisms Described	Potential Relevance for Vestibular Rehabilitation
Spinal level	Vestibulo-spinal pathways; spinal motor neurons	Axonal sprouting; reinforcement of posturo-locomotor synergies	Improved postural stability, antigravity control, and gait adaptation
Supraspinal level	Thalamus, cerebellum, hippocampus, basal ganglia, cortical sensorimotor areas	Distributed network reorganization; multisensory integration; metabolic and synaptic remodeling	Better sensory reweighting, adaptation to complex environments, and improved dynamic balance
Vestibular nuclei level	Ipsilesional and contralesional vestibular nuclei	Restoration of excitability; synaptic remodeling; sensory substitution; reactive neurogliogenesis; neuroimmune modulation	Core substrate of central vestibular compensation and a key target of task-specific rehabilitation
Behavioral/clinical level	Posture, gait, gaze stabilization, visual dependence, symptom tolerance	Adaptation; substitution; habituation; task-specific learning	Functional recovery, reduced disability, and improved participation in daily activities

## Data Availability

No new data were created or analyzed in this study. Data sharing is not applicable to this article.

## Supplementary Materials

The following supporting information can be downloaded at: https://www.mdpi.com/article/10.3390/jcm15166359/s1, Figure S1: Evaluation of the number of publications related to vestibular rehabilitation and pharmacology. Number of publications in the Pubmed database using the keywords “vestibular rehabilitation” and “vestibular pharmacology” since 1950. For over 50 years, vestibular research has focused on the pharmacological approach for the treatment of vestibular pathologies. This trend has been reversed over the last 5 years. In 2023, there were twice as many publications on vestibular rehabilitation as on vestibular pharmacology.

## Abbreviations

The following abbreviations are used in this manuscript:

PV	Peripheral vestibulopathy
CVC	Central vestibular compensation
BDD	Balance disorders and dizziness
