# Supplementary material for: Optimizing Vestibular Rehabilitation: From Neuroplastic Mechanisms to Multimodal Therapeutic Strategies
Source: J Clin Med. 2026 Aug 18;15(16):6359. doi: 10.3390/jcm15166359 (PMC13513125; doi:10.3390/jcm15166359)
Supplement: Supplementary file 1 [file jcm-15-06359-s001.zip › jcm-4408525-supplementary.pdf]

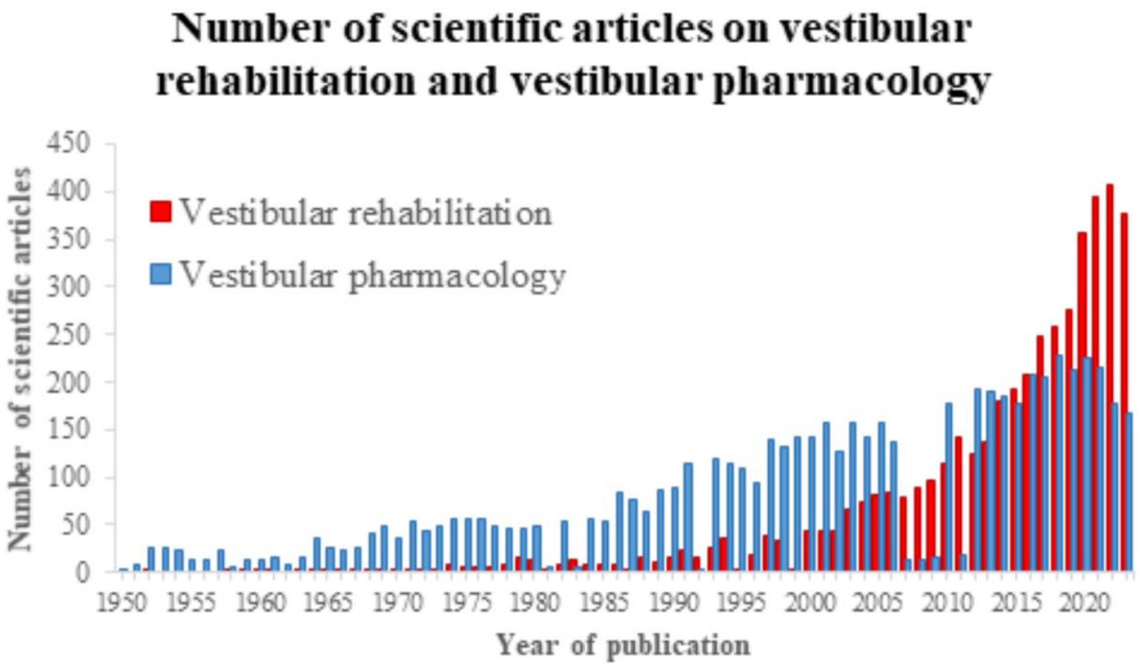

Figure S1. Evaluation of the number of publications related to vestibular rehabilitation and pharmacology.

Number of publications in the Pubmed database using the keywords "vestibular rehabilitation" and "vestibular pharmacology" since 1950. For over 50 years, vestibular research has focused on the pharmacological approach for the treatment of vestibular pathologies. This trend has been reversed over the last 5 years. In 2023, there were twice as many publications on vestibular rehabilitation as on vestibular pharmacology.
